# Paclitaxel-Coated Balloon Angioplasty of Venous Stenoses in Native Dialysis Fistulas: Primary and Secondary Patencies at 6 and 12 Months

**DOI:** 10.5334/jbr-btr.1159

**Published:** 2016-07-22

**Authors:** Nicolas Verbeeck, Jean-Christophe Pillet, Aman Toukouki, Fernand Prospert, Sonia Leite, Xavier Mathieu

**Affiliations:** 1 Centre Hospitalier de Luxembourg, LU; 2 Luxembourg Institute of Health, LU

**Keywords:** Paclitaxel, arteriovenous shunt, renal dialysis

## Abstract

**Purpose:**

When dialysis access stenoses are dilated by noncoated balloons, respective primary and secondary patencies hardly reach 50 per cent and 85 per cent at one year. This study determines the primary and secondary patency rates at 6 and 12 months for venous stenoses treated by paclitaxel-coated balloon (PCB) angioplasty in native hemodialysis accesses.

**Materials and Methods:**

From 2012 to 2014, 70 venous stenoses in 41 patients benefited from PCB angioplasties. The patients’ mean age was 62.5 ± 13.8 years’ standard deviation (SD) with 75 per cent male gender, 31.7 per cent diabetes, and 65.9 per cent arterial hypertension. There were 58.5 per cent forearm fistulas and 41.5 per cent arm fistulas. Primary and secondary patency rates were prospectively established by using the Kaplan-Meier technique and tested by using the log-rank test.

**Results:**

The primary patency rates ± SD were 81.4 ± 4.6 per cent and 60 ± 5.9 per cent at 6 and 12 months, respectively. The secondary patency rates ± SD reached 94.3 ± 2.8 per cent at 6 months and 91.4 ± 3.3 per cent at 12 months.

**Conclusion:**

This study shows an improvement of the primary and secondary patency rates at 6 and 12 months when venous stenoses of native dialysis fistulas are treated by PCB. The indications for PCB, however, remain to be established by larger randomized studies.

## Introduction

As a consequence of the constant aging of the populations, the incidence of end-stage renal disease is steadily rising [1]. When renal transplantation is unavailable or impossible, hemodialysis and peritoneal dialysis remain the main treatment options [2]. For long-term hemodialysis, the European Best Practice Guidelines (EBPG) on Vascular Access and the National Kidney Foundation Kidney Disease Outcomes Quality Initiative (NKF KDOQI) guidelines recommend native arteriovenous fistulas instead of grafts and central catheters because of higher patency rates and lower complications, such as thrombosis and infection, even though native shunts mature less easily and may fail to reach dialysis needs in terms of flow [2,3,4,5,6,7]. The strong rise in chronic dialysis needs has dramatically raised the number of native access creations so that fistula failure becomes a real problem in terms of morbidity and mortality [2].

The main concerns regarding native shunt failures are the anastomotic and venous stenoses that lower the shunts’ flow, which may lead to thrombosis, a complication that seriously impairs the long-term durability of dialysis accesses [7]. Pathogenesis of the stenoses remains incompletely understood, cellular proliferation and cytokine expression being the cornerstones of neointimal hyperplasia [4]. The EBPG and the NKF KDOQI guidelines recommend endovascular treatment as the standard of care, since it has the same efficacy as surgery, at least for venous stenoses, and is simpler and safer to perform [4,5,7,8].

The technique of the endovascular treatment of the venous strictures (i.e. balloon angioplasty) has been extensively described. In short, plain balloons generally allow procedural success, high-pressure balloons help in difficult cases, and the utility of the more expensive cutting balloons remains unproven [6,8,9]. That said, dilation of the stenoses induces local vascular wall damage favouring neointimal hyperplasia and restenosis, impairing primary and secondary patencies [6,10]. Thus, the availability of paclitaxel-coated balloons (PCBs), whose drug action inhibits that neointimal hyperplasia, seems attractive [5,11].

The purpose of our study was to determine the primary and secondary patencies at 6 and 12 months of the venous stenoses treated by a PCB.

## Materials and Methods

We conducted a nonrandomized prospective single-centre study to determine the primary and secondary patencies at 6 and 12 months of native arteriovenous fistulas venous stenoses treated by PCB percutaneous angioplasty. The study protocol was approved by our institutional review board, and written informed consent was obtained. The study began in February 2012 with the first PCB venous angioplasty and ended in November 2014, 12 months after the 70th PCB dilation. Patients less than 18 years old were excluded.

Forty-one patients with a mean age ± SD of 62.5 ± 13.8 years (range 30–83 years), including 31 (75.6%) men and 10 (24.4%) women, benefited from dilations. Thirteen of the 41 (31.7%) patients were treated for diabetes, whereas 27 (65.9%) received antihypertensive drugs.

Forearm fistulas were 24/41 (58.5%) and arm fistulas were 17/41 (41.5%), with 30/41 (73.2%) shunts on the left side. The mean age of the shunts was 67 months (range 10 days to 288 months), and 29/70 (41.4%) of the stenoses had had a previous treatment that was a balloon dilation in 25/29 (86%) of the cases.

Seventy venous stenoses in these 41 shunts benefited from PCB angioplasties. Fifteen of 70 (21.4%) stenoses were discovered via symptoms of shunt dysfunction, as listed in Table 1. The other 55/70 (78.6%) stenoses were identified via an echo-Doppler surveillance program, according to the EBPG and the NKFDOQI recommendations [7,8]. Echo-Doppler criteria for significant stenosis are listed in Table 2. A continuous Doppler waveform in the subclavian vein was considered as an indirect sign of central venous stenosis [12]. For availability reasons, only one thrombosed shunt was included in the study (surgery is preferred in these occurrences in our hospital).

**Table 1:** The indications for PCB dilation (n = 70).


– Difficult punctures	6
– Painful arm swelling	3
– Impaired dialysis flow	2
– Prominent scapular venous collaterals	1
– Lack of maturation	1
– Clotting during dialysis	1
– Fistula thrombosis	1
– One echo-Doppler surveillance program positive criterion at least	55


**Table 2:** Echo-Doppler criteria for significant stenosis.


– Vein diameter < 3 mm	
– Local acceleration ≥ 6 m/s	
– Vein diameter < 50% of the adjacent healthy vein with	a > 20% diminution of the flows and/or a strong flow decrease: < 500 mL (forearm fistula), < 750 mL (arm fistula)


All dilation procedures were performed with patients under continuous electrocardiography and pulse oximetry monitoring; conscious sedation was achieved by an anesthesiologist for fragile or demanding patients; otherwise, 1 or 2 mg of midazolam were slowly administered intravenously before the balloon inflations.

A 5F introducer sheath was inserted in the vein, and fistulography was performed, with or without a tourniquet, to assess the site, the severity and the length of the stricture, and the diameter of the adjacent healthy vein. A 0.035-inch straight, hydrophilic-coated, Terumo guidewire (Terumo Europe, Leuven, Belgium) was used for calibration purposes before angiographic measurements. The venous stenoses’ (i.e diameter reduction compared to the adjacent normal segment) mean length and their mean distance to anastomosis ± SD were 17.2 ± 12 mm and 13.4 ± 12.2 cm. The mean severity of stenoses ± SD was 67.6 ± 9.4 per cent. When required by the size of the PCB, the 5F sheath was exchanged for a 6F one (mandatory for PCB with a diameter of 7 mm).

**Figures 1a and 1b:**
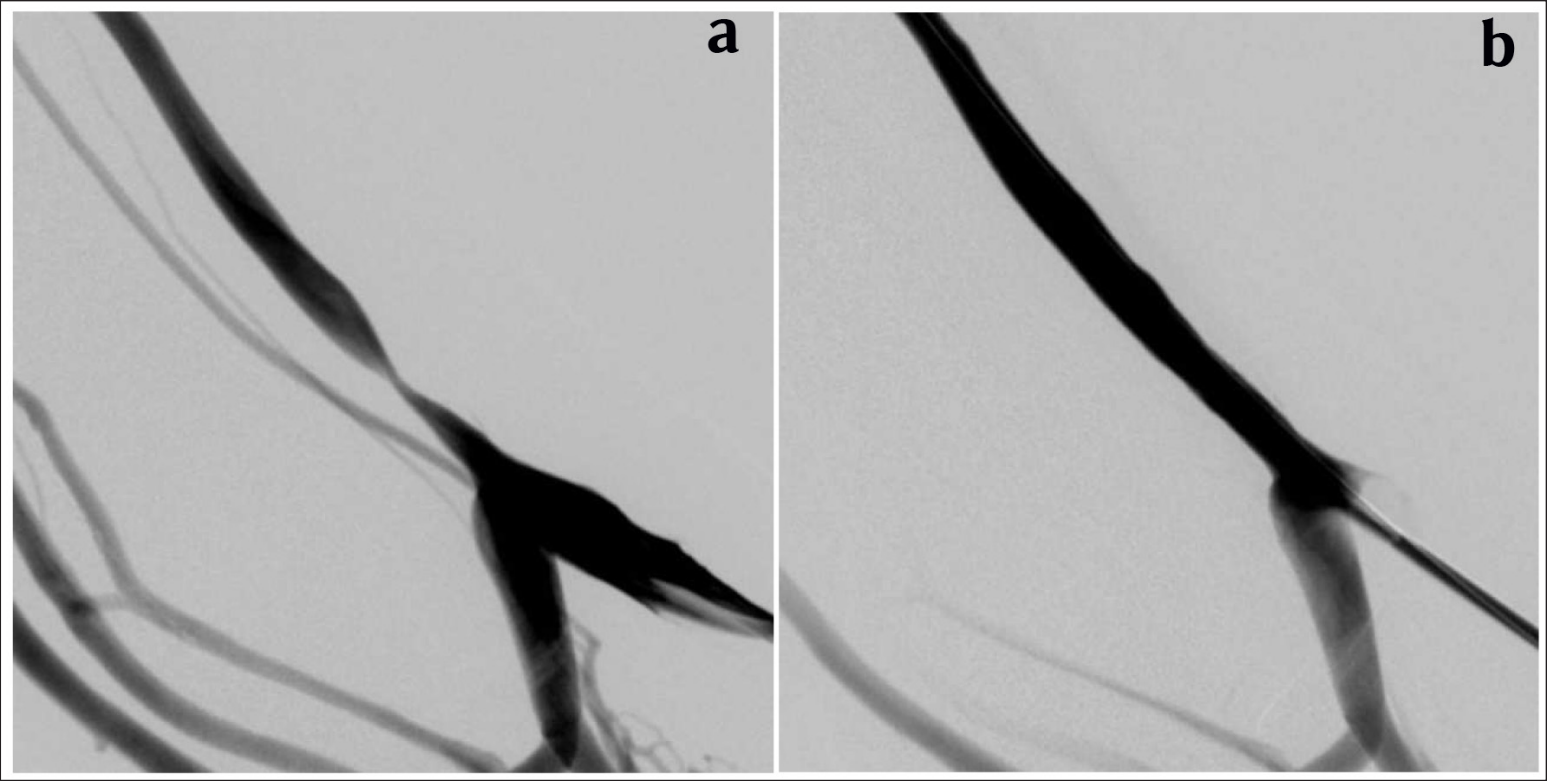
left cephalic vein before (a) and after (b) 7 × 40 mm PCB dilation.

Heparin was administered intravenously in doses up to 3,000 IU according to the patient’s weight. The stenosis was then crossed with the guidewire, using a 5F vertebral catheter (Terumo Europe, Leuven, Belgium) as a torque device. In very complex, anfractuous strictures, thinner guidewires were used. The guidewire was then possibly exchanged for an 0.018 metallic guidewire (Biotronik, Bülach, Switzerland), and the PCB (In.Pact Pacific, Medtronic, Galway, Ireland), with appropriate diameter (5–7 mm) and length (40–60 mm), was then inflated for one minute at prescribed pressure. If a waist persisted on the PCB, overinflation to 18 ATM was applied. A pressure-inflation device (Merit Medical Systems, Sint-Jans-Molenbeek, Belgium) was used for balloon-inflation monitoring in all cases (Figures 1a and 1b).

When the dilation was incomplete, the stenosis being unbreakable or the diameter of the adjacent healthy vein being > 7 mm, we resorted, depending on the case, to cutting balloons (Boston Scientific, Galway, Ireland) (five cases) or to using larger high-pressure balloons (Biotronik, Bülach, Switzerland) of 8 or 9 mm in diameter (seven cases).

For each patient, an echo-Doppler was performed 10 days after the procedure to check fistula flows in order to collect basis values (no overflow was noticed). Then, even without any symptom of dysfunction of the shunt, the patients benefited from echo-Doppler controls at 3, 6, and 12 months. If, in the 12-month period, the fistula showed any sign of significant stenotic resurgence, be it in clinical or echo-Doppler terms, the shunt was dilated again. Stenoses at another site than the previously dilated one were treated by conventional, high-pressure, or cutting balloons, and restenoses at the same site benefited from PCBs. The former were excluded from the secondary patency rates, whereas the latter were included.

Primary patency for a dilated stenosis was defined as uninterrupted patency of the treated site after PCB dilation, and secondary patency was defined as patency of the treated stenosis following endovascular reintervention with a PCB at the same site. We did not resort to the recommended standard of assisted primary patency since we wanted to compare our results to the main studies, using noncoated balloons, which mostly mentioned primary and secondary patencies [13]. We considered patency failure in case of ineffective dialysis, when a suggestive clinical symptom (e.g. painful arm swelling) appeared, or when an echographic or a Doppler sign of significant stenosis was discovered.

Primary and secondary patency rates for venous stenoses after PCB dilation were established by using the Kaplan-Meier technique and tested by using the log-rank test. Predictors of dilated stenosis patency, such as the length of the stenosis or the age of the fistula (cf. infra), were determined by using a Cox proportional hazards regression model with clinical and anatomic variables. Variables identified in the univariate Cox model with P < 0.2 were included in the multiple-regression model by using a stepwise regression. A two-sided P value of less than 0.05 was considered to indicate a statistically significant difference. Statistics calculation was carried out by the Luxembourg Institute of Health with the SAS version 9.4 software.

## Results

An initial success rate (residual stenosis < 30%) of 58/70 (82.9%) due to PCB waist or insufficient diameter of the PCB rose to 100 percent with additional procedures. The mean duration ± SD of a dilation was 19.1 ± 8.3 min. During the procedures, five cases of venous extravasation occurred at the site of dilation and were easily controlled by balloon inflation at 2 ATM and/or external compression. Spasms and elastic recoils occurred, but none needed to be treated. One case of infection of the puncture site responded well to broad-spectrum antibiotic therapy.

During follow-up, three shunts were abandoned because early (one to four months) and unexpected extensive thrombosis occurred, and complex endovascular treatments were considered too heavy due to the patients’ poor clinical status (very old age and/or severe cardiac deficiency); these patients were dialysed via central catheters. The three fistulas comprised five PCB-dilated stenoses that were included in the data. Three patients died, unrelated to the procedures, and were censored.

**Figure 2:**
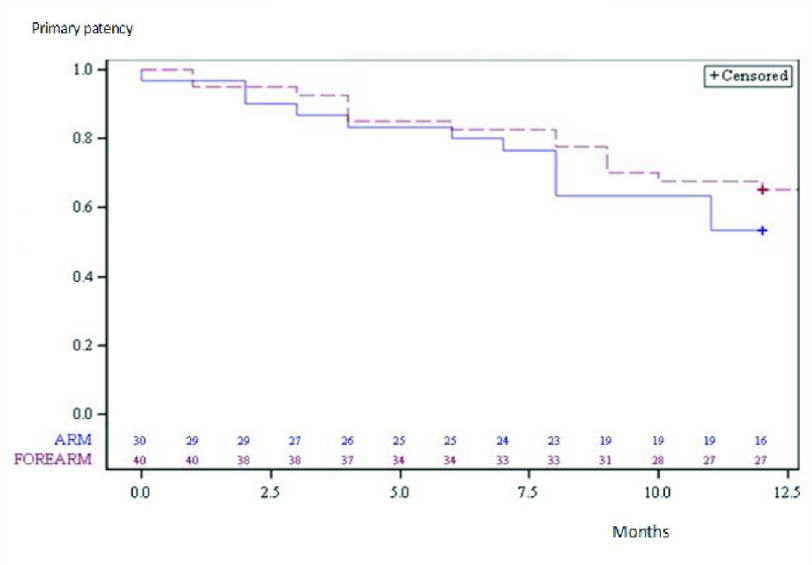
12-month Kaplan-Meier curves of dilated venous stenoses estimated primary patency of the forearm and arm fistulas.

At 6 and 12 months, respectively, primary patency rates ± SD for the dilated venous stenoses were 81.4 ± 4.6 per cent and 60 ± 5.9 per cent. For the arm fistulas, primary patency rates were 80 ± 7.3 per cent and 53.3 ± 9.1 per cent at 6 and 12 months, whereas, for the forearm fistulas, they were 82.5 ± 6 per cent and 65 ± 7.5 per cent (Table 3). No significant difference was noted between the Kaplan-Meier curves of the forearm and the arm accesses (P = 0.328) (Figure 2).

**Table 3:** Primary and secondary patencies ± SD, at 6 and 12 months, of the PCB-dilated stenoses.

	All fistulas		Arm fistulas		Forearm fistulas	
	6 months	12 months	6 months	12 months	6 months	12 months

Primary patency	81.4 ± 4.6%	60 ± 5.9%	80 ± 7.3%	53.3 ± 9.1%	82.5 ± 6%	65 ± 7.5%
Secondary patency	94.3 ± 2.8%	91.4 ± 3.3%	90 ± 5.5%	90 ± 5.5%	97.5 ± 4.2%	92.5 ± 4.2%

The only significant factor negatively impacting venous primary patency was the length of the stenosis (hazard ratio (HR) per centimetre = 1.038, P = 0.008): the longer the stenosis, the shorter the primary patency (Table 4).

**Table 4:** Primary and secondary patency potential influencing factors.

Potential influencing factors	Primary patency		Secondary patency	
	P-value	Hazard ratio	P-value	Hazard ratio

– Gender of the patient	0.644		0.699	
– Age of the patient	0.180		**0.005**	0.903
– Arterial hypertension	0.454		0.201	
– Diabetes	0.807		0.883	
– Type of the arteriovenous fistula (arm/forearm)	0.525		0.956	
– Side of the arteriovenous fistula	0.417		0.577	
– Age of the arteriovenous fistula	0.063		0.164	
– Previous intervention on the stenosis	0.805		0.663	
– Percentage of the stenosis	0.202		**0.037**	1.106
– Length of the stenosis	**0.008**	1.038	0.437	
– Distance of the stenosis from the anastomosis	0.257		0.167	

**Figure 3:**
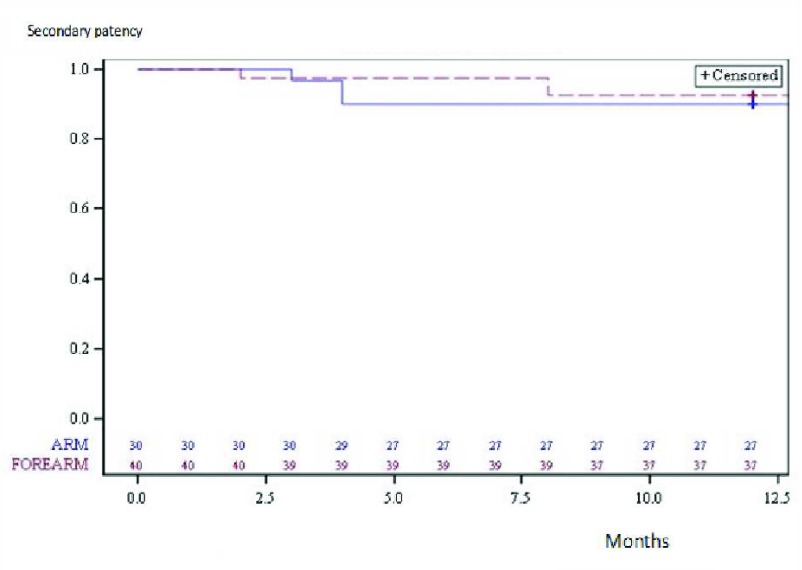
12-month Kaplan-Meier curves of dilated venous stenoses estimated secondary patency of the forearm and arm fistulas.

For all the dilated venous stenoses, secondary patency rates ± SD, at 6 and 12 months, were 94.3 ± 2.8 per cent and 91.4 ± 3.3 per cent. For the arm fistulas, they were 90 ± 5.5 per cent and 90 ± 5.5 per cent, whereas, for the forearm fistulas, they were 97.5 ± 4.2 per cent and 92.5 ± 4.2 per cent (Table 3). For the primary patency, the difference between the Kaplan-Meier curves of the forearm and the arm shunts was not significant (P = 0.699) (Figure 3).

Stepwise regression was also performed for secondary patency. The only factors with a significantly negative impact on the secondary patency were the younger age of the patient (P = 0.005) and the higher stenosis severity (P = 0.037) (Table 4>).

## Discussion

In this study, we determined the primary and secondary patencies at 6 and 12 months, that is, 84.1 per cent, 60 per cent, 93.4 per cent, and 91.4 per cent, respectively, of native arteriovenous fistulas stenoses that were dilated by PCB. The patency rates we observed compare favourably to other series using noncoated balloons, even though our echo-Doppler surveillance program, which allows early detection of the stenoses, has possibly lowered the patency percentages. For example, one publication presented a primary patency with a conventional balloon at 12 months reaching 48.5 per cent, whereas, in another article, also using plain balloons, primary patency at 12 months was 41.4 per cent [4,10]. However, comparison of our work with other investigations remains somewhat complicated because of the differences in the studies’ protocols in terms of methods of detection of the stenoses and their quantification, among others [14]. We did not find any significant difference between the primary and secondary patency curves of the forearm and versus the arm fistulas, which contradicts previous studies, but may be another effect of the paclitaxel.

The greater length of the stenosis was the only negative predictor of primary patency, whereas the higher severity of the stricture and the younger age of the patient were the sole unfavourable predictors of secondary patency. As far as secondary patency significant predictors calculation is concerned, the small number of restenoses (22) may have biased the results. Classical predictors such as gender, diabetes, or fistula age did not statistically modify primary or secondary patencies. This was also noted in one study [3]. Hypertension, previous treatment, distance to anastomosis, clinical detection of the stenosis, or their detection via an echo-Doppler surveillance program were not statistically significant predictors of patency either. Thus, it might be reasonable to suppose that the longer the stenoses, the greater the benefit of PCBs. However, our study is not capable of providing a formal answer to this question.

The good efficacy of the PCB results in the inhibition of neointimal hyperplasia by paclitaxel, a mitotic inhibitor. The problem is that engineering has a cost: a PCB is much more expensive (up to three times the price in Luxembourg) than a conventional or a high-pressure balloon, and it may, quite logically, be inflated only once. Moreover, some additional dilation procedures must be performed with high-pressure balloons, cutting balloons, or balloons exceeding the diameter of 7 mm (maximal diameter of the In.Pact Pacific).

Should PCBs only be used in selective cases of long venous stenosis? Should they be kept for restenosis? As early unexpected thrombosis occurred in three shunts after PCB dilation, should short-term anticoagulation be considered? Since we recognize that our series is small, implying possible bias, and particularly for the secondary patency section, concerning 22 restenoses only, further randomized, multicentric studies, with larger cohorts of patients are needed to refine the indications for PCBs.

## Conclusion

PCBs appear to improve the primary and secondary patency rates after the treatment of venous stenoses in native hemodialysis fistulas. The main problem remains in their much higher cost, possibly indicating that they should be indicated for selected cases. Further larger and randomized studies must be launched to define the best indications of this expensive device more precisely.
